# Human Excreta and Food Waste of a Typical Rural Area in China: Characteristics and Co-Fermentation

**DOI:** 10.3390/ijerph19084731

**Published:** 2022-04-14

**Authors:** Jean Joël Roland Kinhoun, Ao Li, Minghuan Lv, Yunpeng Shi, Bin Fan, Tingting Qian

**Affiliations:** 1 State Key Laboratory of Environmental Aquatic Chemistry, Research Center for Eco-Environmental Sciences, Chinese Academy of Sciences, Beijing 100085, China; jrkinhoun@live.com (J.J.R.K.); liao_rcees@163.com (A.L.); lvmh_1994@163.com (M.L.); ypshi_st@rcees.ac.cn (Y.S.); fanbin@rcees.ac.cn (B.F.); 2 Water Pollution and Control Department, University of Chinese Academy of Sciences, Beijing 100049, China

**Keywords:** rural environment sanitation, toilet wastewater, food waste, property study, co-fermentation

## Abstract

Human excreta (HE) and food waste (FW) are the primary contaminants in rural regions. Prior to treating these contaminants, mastering their properties is required. In this study, the characteristics of the HE leaving the body and FW leaving the kitchen to the subsequent respective fermentation were studied. Moreover, two kinds of co-fermentation processes for HE and FW were also investigated on the basis of mastering the properties. The results showed that, for a healthy adult, fresh feces, urine, and FW produced were about 163 g/cap/d (57.3 gCOD/cap/d), 1.6 L/cap/d (6.7 gN/cap/d), and 250 g/cap/d (35.0 gCOD/cap/d), respectively. In HE, about 75% of nitrogen and phosphorus were contained in urine. It takes at least three days for crushed FW discharged via water flushing to settle completely, and the COD removal efficiency after precipitation was around 75%. Mixing HE with FW after discharge, i.e., the initial unit of the process was 20% more efficient in fermentation than mixing after the respective pre-fermentation. This paper presents the characteristics of HE and FW and provides the optimized co-fermentation process, which provides technical support for the realization of environmental sanitation in rural areas.

## 1. Introduction

The provision of modern environmental sanitation services for rural areas is essential content to achieve the goal of social modernization in China. Establishing a clean living and ecological environment is an essential component of modern environmental sanitation services. Therefore, the Chinese government raised the “Rural Toilet Revolution”, which aims to improve the residential and ecological environment, as well as the sanitary discharge and treatment of household pollutants (human excreta, food waste, and washing wastewater) [1]. Due to the weak economic fundamentals, partial rural areas cannot afford adequate sanitation systems to treat all domestic pollutants [2]. For such areas, taking the initiative to solve the sanitary discharge of human excreta (HE) and food waste (FW), two primary sources of domestic pollution in rural areas may quickly realize the modernization of environmental sanitation [3]. Before choosing the technology to treat HE and FW, mastering the properties and discharging characteristics of the two sources is necessary.

Previous studies have reported that the property of toilet wastewater (TW, meaning HE mixed with flushing water) and FW vary highly, often on the basis of average values and habits [3,4]. Generally, TW is characterized by chemical oxygen demand (COD, 16.5–46.0 g/cap/d), ammonia nitrogen (NH_3_–N, 3.75–12.72 gN/cap/d), and total nitrogen (TN, 5.35–16.40 gN/cap/d), with 25–40 L/cap/d production [5,6]. Moreover, the content of FW pollutant range is about 35.8–71.9 gCOD/cap/d, 3.83–8.74 gNH_3_-N/cap/d, with 11–490 g/cap/d production [7,8,9]. Usually, the measurement method of these emissions involves setting up a storage tank first and discharging TW or FW into the storage tank and then measuring the sewage quality in the storage tank to obtain the pollutant emissions. However, these results can only reflect the sewage quality pretreated by the precipitation or screen process, and it is difficult to obtain the real characteristics of HE and FW on the basis of this method [3]. 

Moreover, many papers have reported detailed investigation on the anaerobic co-digestion TW and FW for methane production and recycling [5,10]. However, these anaerobic reactors do not fit in rural areas because of the strict anaerobic environment and reasonable maintenance requirements. These technologies need a certain scale (which means centralized collection) and satisfactory operation and maintenance management (meaning high maintenance costs). These two points are difficult to meet under the premise of a low economic development level and waste classification comparison for the characteristics of scattered living in rural areas in China. Therefore, under the current situation, mixing food waste into septic tanks is a cost-effective means of environmental sanitation treatment in rural areas of China.

Septic tank is the most commonly used method as an onsite treatment system for domestic wastewater in many regions because of its low price and simple maintenance [11]. However, few investigations have reported the synergistic treatment of TW and FW in septic tanks at an onsite level. Moreover, the co-treatment of TW and FW has several positive effects [12]. One of the factors affecting the efficiency of co-fermentation is acid–base neutralization. An appropriate pH range is essential for the hydrolysis reaction in the anaerobic process, especially for substrates with easily degradable organic components such as FW, and often cause low-pH inhibition in the process [13]. Whereas the TW has high alkalinity, it can improve the buffer performance and supplement trace elements to help anaerobic digestion [14] (Gao et al., 2020). Another positive effect is that HE contains large numbers of intestinal microbes, serving as an inoculant in the co-digestion process of HE and FW, which would accelerate the start-up of the co-fermentation reaction [15,16]. 

Therefore, the purpose of this research was to study the characteristics of two major domestic pollutants, HE and FW, and obtain the basic properties and emission rules for the two kinds of contaminants. At the same time, on the basis of the principle of high efficiency and low cost, septic tank technology was selected to treat the mixture of TW and FW. The advantages of collaborative fermentation treatment were elaborated, and the effects and parameters of the co-fermentation process were obtained, providing theoretical support for its practical engineering application.

## 2. Materials and Methods

### 2.1. Sample Collection

#### 2.1.1. Feces, Urine, and Flush-Toilet Wastewater

Feces and urine were collected from two healthy adults, directly using waste bags and bottles with no flush water added. Flush-toilet wastewater was collected from a TW collection tank, which contains wastewater from a household flush toilet of six adults in Rugao City, Jiangsu Province, China.

#### 2.1.2. Food Waste

FW used in our experiment refers to cooked leftovers and raw food waste. It was collected from the same aforementioned household and represented 80% raw and 20% cooked material, consistent with the eating habits of residents in China [17]. It was a mixture of potatoes, pasta, bread, vegetables, salad, rice, noodle, vegetables, fish, and meat. After bones and non-food materials were removed, a certain weight of FW was wet with a certain volume of tap water; then, a food waste blinder was used to grind the weighed wet food waste to generate a homogeneous mixture with a size less than 2 mm by adding the measured amount of tap water step by step. Finally, we mixed the grinded food waste with all the weighed tap water to form the food waste crushing solution used in our experiments. The mixture of food waste and water followed the ratio of 1 g (wet weight)/10 mL (volume). The “precipitated solids” are therefore the precipitates obtained by the sedimentation of the food waste.

### 2.2. Experimental Design

#### 2.2.1. Fermentation of Feces and Urine

Flush condition toilets were investigated by mixing feces and urine with water. The toilet condition was based on the real situation of toilet utilization; the experiment of fermentation was simulated as realistically as possible. The toilet design was one-time feces (about 150 g) plus six liters of flush water, one-time urine (about 500 mL) plus six liters of water, and one-time feces plus five to six times urine and about 36 L of water. The samples were then tested every hour to determine the parameters (volume, mass, total solid (TS), volatile solid (VS), chemical oxygen demand (COD), soluble chemical oxygen demand (SCOD) pH, total nitrogen (TN), ammonia (NH_3_-N)).

#### 2.2.2. Fermentation of Food Waste

In this section, we investigated the fermentation process of FW at different temperatures. Two beakers were fed with one-liter FW waste crushing liquid, then kept at 4 °C and 20 °C temperatures. These batch experiments were run continuously for 20 days, different parameters were monitored, and sediment quantity and the scum were measured optically. The sediment merely was solid particles that did not get filtered away during the preparation of the crushing solution.

#### 2.2.3. Co-Digestion of Toilet Wastewater and Food Waste

**Figure 1:**
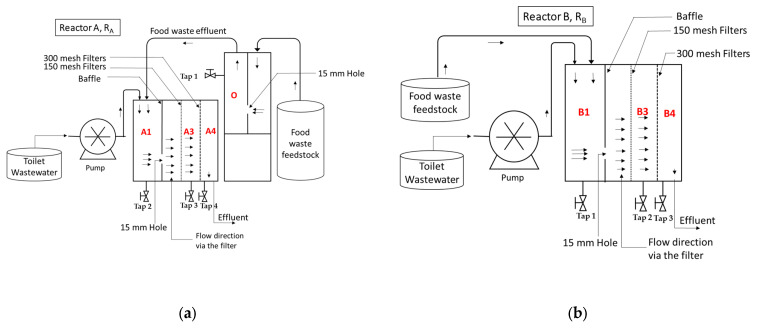
Scheme of experimental set-up for (**a**) two-stage digestion FW and TW (Reactor A, R_A_) and (**b**) one-stage digestion FW and TW (Reactor B, R_B_) reactors. A1, A3, A4: the first, third, and fourth compartment of reactor A, respectively; likewise, for B1, B3, and B4 for reactor B; O: the compartment of single digestion FW unit for reactor A.

The anaerobic co-fermentation of FW and TW was conducted in two-stage and one-stage digestion with an enhanced septic tank. These reactors were made from polyester PVC (Figure 1). The two-stage digestion FW and TW (Reactor A, R_A_) consisted of a stabilization unit that digests only FW without any additional process, followed by a section treating TW and the acidified effluent from the stabilization unit of FW.

One-stage digestion FW and TW (Reactor B, R_B_) consists of one reactor treating FW and TW together without pretreatment. The performance was compared between two-stage and one-stage reactors. Both reactors had the same working volume of 16.5 L.

**Figure 2:**
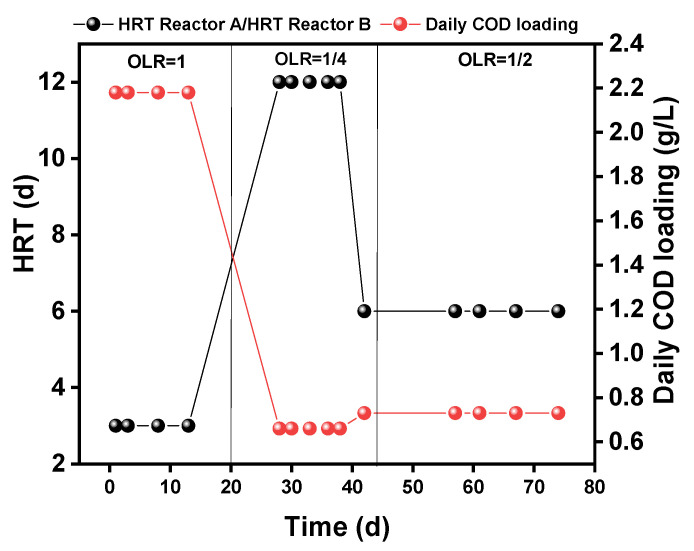
Operational scheme for the laboratory scale reactors. ORL: organic loading rate.

All the systems were installed in a temperature-controlled room (at 24 °C); the reactor was fed every day but was not fed with sludge to enhance the growth of bacteria. The different operating conditions of daily loading and HRT are shown in Figure 2. The HRT of the reactor at the beginning of the operation was 3 days; then, after 20 days operation, the HRT was increased to 12 days for 25 days operation, then decreased to 6 days HRT until the end of the operation.

On the basis of the average 6 L TW/cap/time flushing and 250 g FW/cap/d in China, the feedstock used in this study was prepared with FW/TW = 250 g of FW/cap/day and 36 L TW/cap/day, then was mixed to feed each reactor. The reactor was fed continuously with TW at different times (8:30 am, 10:30 am, 2:30 pm, 4:30 pm, 7:30 pm, 9:30 pm) per day via connection to a neighboring building and one time (12:30 pm) by FW per day.

### 2.3. Analytical Methods

Raw FW, TW, and effluent from the reactors were collected and analyzed daily. The pH was measured every day with the pH meter (HQ30D53000000-PHC10103, Hach company, Loveland, CO, USA). The COD and soluble chemical oxygen demand (SCOD) concentrations were measured using the reagent bottle method (COD reagent 20–1500 mg/L, Hach company, Loveland, CO, USA). A 0.45 µm filter was used to filter the samples for SCOD, VFA, and NH_3_-N before determination. The different VFA were determined using a Agilent Technologies 7890B gas chromatograph (Agilent Technologies, Inc., Wilmington, DE, USA) equipped with a flame ionization detector (FID) and a DB-FFAP column (Agilent Technologies, Inc. Wilmington, DE, USA). The total solid (TS) and volatile solid (VS) compounds were analyzed by using the standard methods of [18]. The concentrations of total phosphorus (TP), TN, and NH_3_-N were performed according to the standard methods [19]. 

We checked the variability of the data following the reactor by doing a one-way analysis of variance. In case of significant variability (*p* < 0.05), we further performed a mean comparison following the least significant difference (LSD) test. Thus, we clearly highlighted the means difference with the corresponding letter at upper position on the bar graph.

### 2.4. Calculation

The COD removal efficiency was calculated as follows:

(1) $$COD\;removal\;efficiency=\frac{CODinfluent-CODeffluent}{CODinfluent}\times 100\%$$

where *COD removal efficiency* (%); *COD influent* = COD fed to the reactor (g); *COD effluent* = COD discharged from the reactor (g).

## 3. Results and Discussion

### 3.1. Characteristics of Feces and Urine

#### 3.1.1. Properties of Feces and Urine

The property of fresh feces and urine was investigated using the samples produced by six healthy adults living on a typical farm. The collection of fresh feces and urine lasted for three consecutive days. Table 1 shows the characteristic properties of fresh feces and urine. As shown in Table 1, the mass of adult feces discharged per day was about 163 ± 3 g (wet weight), its water content was about 76.9%, and more than 90% of the solid feces was organic matter. The dissolved organic matter (DOM) only accounted for 18.9%. The urine discharged per adult per day was about 1.6 ± 0.2 L, and the density of fresh urine was about 1.0 ± 0.1 g/mL, in which the content of DOM accounted for 94% of the total organic matter.

**Figure 3:**
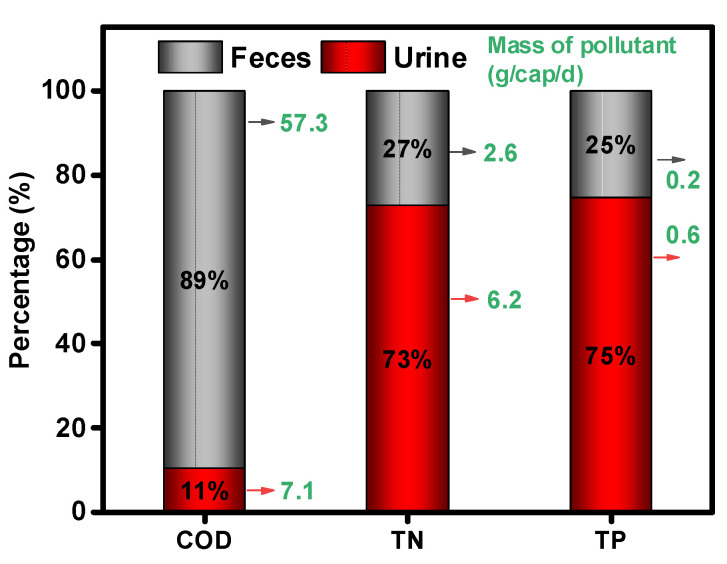
The contribution of excreta and urine for COD, TN, and TP contained in human excreta. TN—total nitrogen, TP—total phosphorus.

Figure 3 presents the contribution of feces and urine for COD, TN, and TP in HE. It was found that urine is the predominant nitrogen and phosphorus contributor with 73% and 75%, respectively. It was reported that urine contains the largest proportion of nitrogen at about 80–90%, and phosphorus at 50–65%, which is consistent with our results [20,21]. 

#### 3.1.2. Respective and Synergistic Fermentation of Feces and Urine

**Figure 4:**
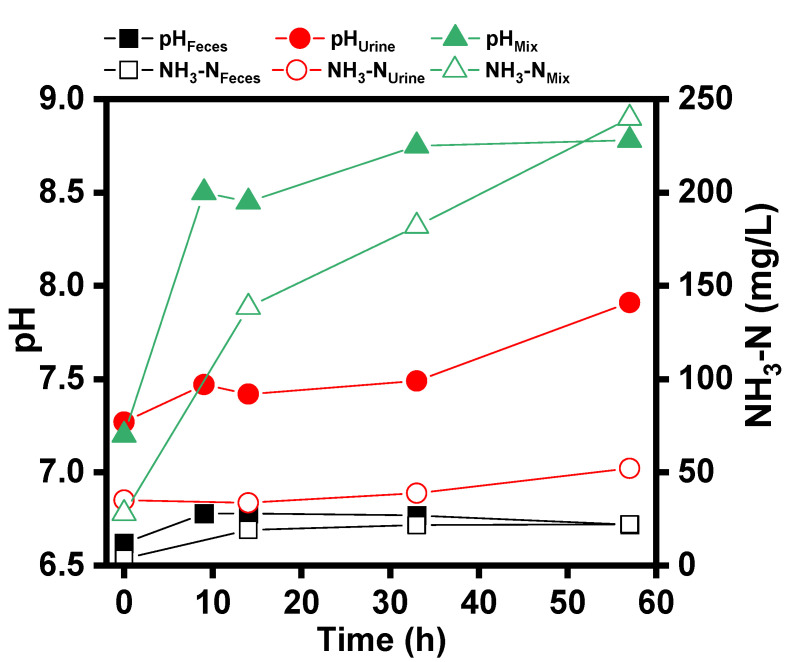
pH and NH_3_-N variation for feces, urine, and their mixture.

Figure 4 shows the changes in pH and NH_3_-N over time for the fresh feces, urine, and feces and urine mixture (Mix) after being discharged from a flush toilet (6 L/flush). As shown in Figure 4, the changes in pH and NH_3_-N were not significant for both feces and urine flush discharges when stored separately. For feces, the pH increased from 6.6 to 6.7, and the NH_3_-N concentration increased from 3.7 mg/L to 22.2 mg/L after about 60 h of storage alone. Similar variation was examined for urine, with the pH increasing from 7.2 to 7.9 and the ammonia nitrogen increasing from 35.2 mg/L to 52.2 mg/L. However, when discharged feces was mixed with urine, the pH and ammonia concentration of the mixture changed significantly. During the period of 14 h after mixing, the pH increased from 7.2 to 8.4, and the ammonia nitrogen concentration increased from 28.1 mg/L to 138.6 mg/L.

The difference in the variation of pH and NH_3_-N concentration before and after mixing could be related to the decomposition of urea in fresh urine [22]. It has been known that the decomposition of urea produces bicarbonate and ammonia nitrogen. Urea hydrolysis will produce carbonate and bicarbonate, which will produce hydroxyl after hydrolysis, so as to increase the pH value of the solution [23]. However, the decomposition of urea requires the catalysis by urease. The fresh urine is pure without urease-producing microorganisms, and fresh feces contains a large number of intestinal microorganisms capable of producing urease [24]. Therefore, when fresh urine and feces were mixed, the pH and ammonia concentration of the mixture changed significantly under the catalysis of enzymes secreted by microorganisms in feces.

### 3.2. Characteristics of Food Waste

#### 3.2.1. Properties of Food Waste

The FW average mass produced by the family in our study was about 250 g/cap/d, with a contaminant mass of 35 gCOD/cap/d, 0.87 gN/cap/d. Other properties such as moisture (average 87%) and organic matter content (about 89%) have been found. For instance, it was reported elsewhere that FW production for Europe and North America is estimated to be 223–447 g/cap/d [25,26], and for Korea at 260 g/cap/d [27]. 

#### 3.2.2. Fermentation of Food Waste

**Figure 5:**
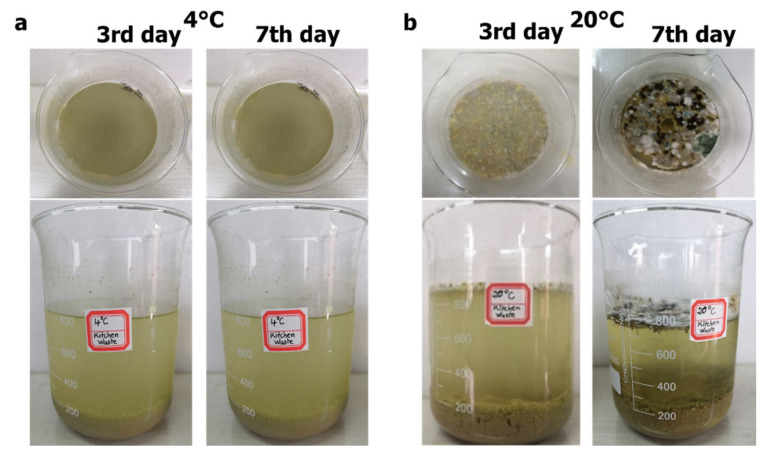
Appearance variation of samples at different temperatures and at different days: (**a**) 4 °C, (**b**) 20 °C.

Figure 5 shows the apparent changes of the crushing solution of FW stored for 3 and 7 days at the environmental temperatures of 4 °C and 20 °C. As shown in Figure 5, for 1 L crushing solution, the volume of the precipitated solids in the beaker was about 200 mL at both 4 °C and 20 °C on days 3 and 7. On the one hand, it shows that the precipitated volume of the crushed liquid solids was about 20% of the total volume at this waste-to-tap mixing ratio (1 g:10 mL). On the other hand, it proves that the low temperature did not affect the precipitation process of the crushed liquid. As shown in Figure 5b, for the supernatant at 20 °C, scum was observed during the experiment, and fungal communities grew on the surface layer. However, there was no significant phenomenon on the surface layer for the supernatant at 4 °C. The low temperature might have limited the process of fermentation and gas production of the sediment at the bottom of the beaker. Therefore, the surface layer of the supernatant at low temperature did not form scum due to the uplifting of the bottom sediment, which led to no fungal community formation on the surface at low temperature. The scum consumption of fungal communities can be regarded as an important process to decompose the solids in the crushed liquid.

**Figure 6:**
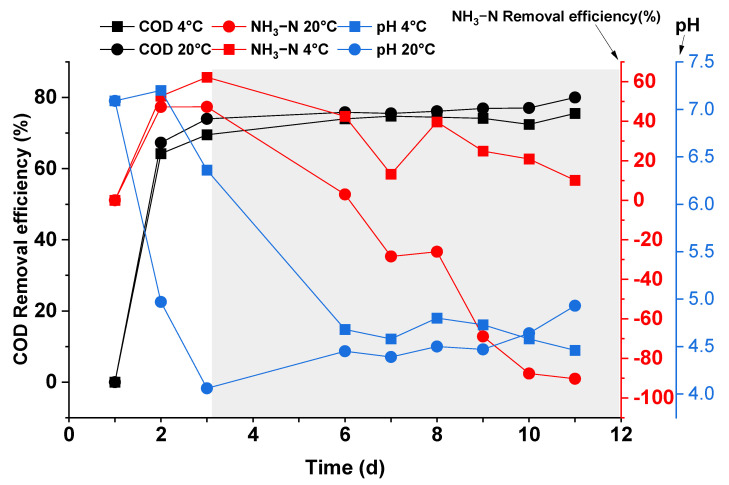
Variation of supernatant in terms of COD, NH_3_-N removal efficiency, and pH during the experiment.

Figure 6 shows the changes in pH with respect to the removal efficiency of COD and NH_3_-N during the experiment. As shown in Figure 6, the pH gradually decreased from the initial value of 7.2 and stabilized at 4.5. The pH for the beaker at 4 °C decreased from 7 to 4.5 at day 6, then slightly varied from day 6 to 12. In the case of 20 °C, the pH decreased from 7 to 4.0, then increased from pH 4 to 5 between days 3 and 12 and kept an increasing trend. The change of the pH is very slow for both reactors.

Moreover, the low temperature was shown to have no significant effect on the COD removal efficiency of the crushing liquid. The COD removal efficiency increased at 70% on the third day, and from that day, the change was very slow for the experiment in both groups. In the initial phase (first three days), the COD removal from the food waste comminution was mainly by sedimentation in the absence of inoculated sludge inside the beaker. As the experiment progresses, the beaker surface scum and the bottom sediment forming indicate that bacterial fermentation occurred in the sample (after respiration ceased due to O_2_ depletion), which naturally led to a decrease in COD, thus achieving this COD removal.

There are two processes involved in the variation of NH_3_-N removal efficiency. The first process is the rising stage relevant to the precipitation and supernatant of solid organic nitrogen. The NH_3_-N removal efficiency during the rising stage of the two groups was similar; the second process is the descending stage, which could be related to the hydrolysis and acidification of solid organic nitrogen to form dissolved NH_3_-N. Because higher temperature will accelerate the rate of hydrolysis and acidification, the removal efficiency of NH_3_-N at 4 °C increased faster than 20 °C. The trends slowly change and stayed positive until the 12th day.

Similarly, the low temperature significantly reduced the fermentation rate of the FW crushing liquid, which in turn affected the changes of pH and NH_3_-N in the supernatant.

The same amount and nature of food waste crushing liquid (Supplementary Materials Figure S4), added the same test days, formed a bottom sediment with approximately the same apparent thickness at the third day, and the bottom sediment formed was the sedimentation of larges particles (Figure 5). After several days of operation (at the seventh day), the thickness of the sediment stayed the same. In addition, the COD removal efficiency of the two beaker reactors in the first three days were similar under different temperatures (Figure 6). Therefore, it can be explained that 4 °C and 20 °C had little effect on the sedimentation process of kitchen waste crushing liquid. The low temperature had no significant effect on the settling rate of solids in the FW shredder. When the FW was mixed with water in the ratio of 1 g (wet weight)/10 mL (volume), three days was the necessary period to fully settle the solids in the shredded solution.

### 3.3. Toilet Wastewater and Food Waste Co-Stabilization Reactors

In order to compare the performance of two different processes for the co-fermentation, two reactors, Reactor A (R_A_, two-stage digestion of FW and TW) and Reactor B (R_B_, one-stage digestion of FW and TW), were operated in parallel at similar operating conditions. The two reactors operated at the same temperature of 24 °C, applying the same feedstock of food waste and toilet wastewater. In addition, the HRT was same for both reactors. The process of co-fermentation mainly involves the anaerobic fermentation reaction, e.g., hydrolysis, acidification, and methanogenesis, and therefore several relevant factors of SCOD, COD, NH_3_-N, TN, VFA, and pH were monitored [28].

#### 3.3.1. Comparison between Reactor A and Reactor B

**Figure 7:**
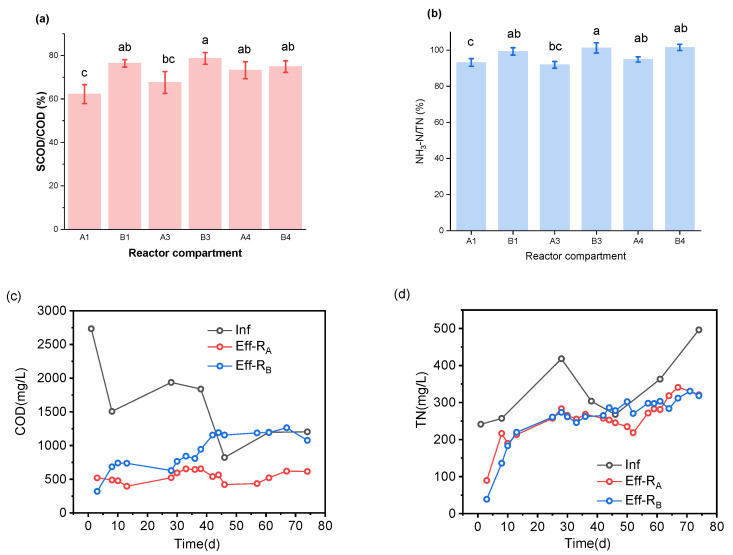

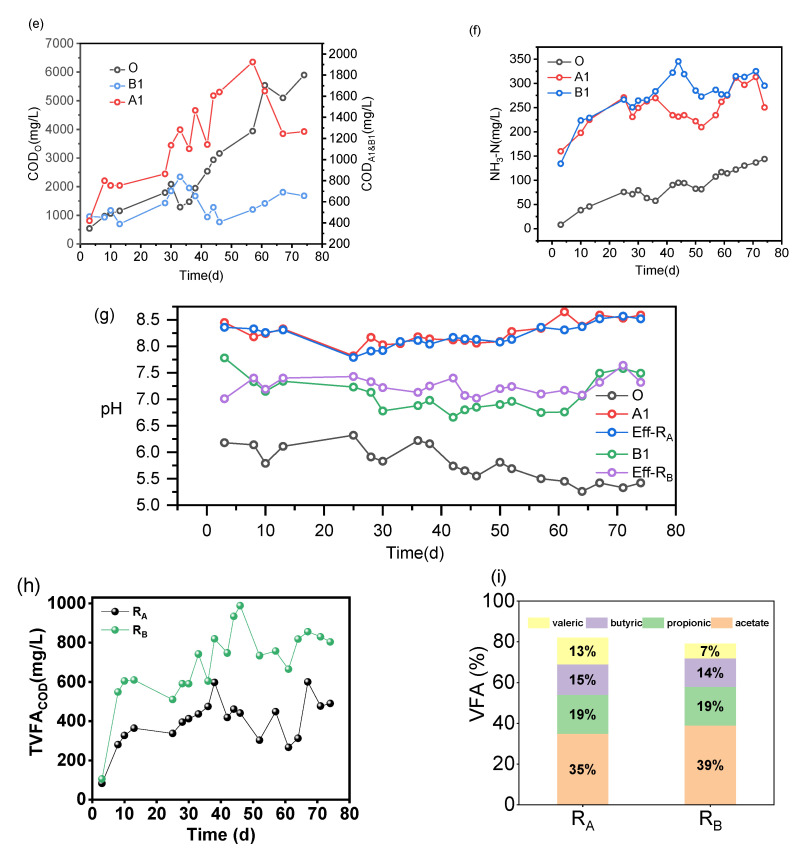
Fermentation effect of two reactors, (**a**) SCOD/COD and (**b**) NH_3_-N/TN, of the last two compartments of R_A_ and R_B_. (**c**) COD and (**d**) TN concentration in the influent and effluent of two reactors. (**e**) COD and (**f**) NH_3_-N concentration in compartment-O for single digestion FW and the first compartment unit co-fermentation (A1 and B1) of R_A_ and R_B_. (**g**) pH variation inside the two reactors. (**h**) Variation of TVFA concentration of R_A_ and R_B_ and (**i**) VFA component features in the effluent of two reactors. A1, A3, A4: the first, third, and fourth compartments of reactor A, respectively; likewise for B1, B3, and B4 for reactor B. Eff-R_B_: the effluent of reactor B, likewise for Eff-R_A_; Inf: influent; O: the compartment of a single digestion FW unit for reactor A; TVFA_COD_: COD equivalent of total VFAs; R_A_: Reactor A, two-stage digestion of FW and TW; R_B_: Reactor B, one-stage digestion of FW and TW.

Figure 7 shows the fermentation of two kinds of co-stabilization processes. As shown in Figure 7a, the hydrolysis rate of R_B_ was a little faster than that of R_A_, roughly judging by the statistically difference between A1 and B1, and A3 and B3 in the ratio of SCOD to COD (SCOD/COD). The paired reactor compartment mean comparison showed (Supplementary Materials Figure S1) a significant difference between A1 and B1, and A3 and B3 for (SCOD/COD) with a *p*-value = 0.02394. On the basis of the significant difference between them (Supplementary Materials Figure S1), we can indicate that the hydrolysis rate in A1 was less than in B1 according to the ratio (SCOD/COD).

As shown in Figure 6e, the COD variation in the mixing unit (A1, B1) of TW and FW was different. COD concentration in compartment A1 trend was influenced by the increasing COD in compartment O, singly fermenting FW. Indeed, the compartment A1 was able to balance the impact of that action caused by the compartment “O’’ variation. In addition, the effluent from compartment ‘’O’’ was combined with TW influent in the compartment A1, promoting a better hydrolysis balance for the reactor. As shown in Figure 7c,d, under the premise of the same feedstock, the COD concentration in the effluent of R_B_ was ≈2 times higher than that of R_A_.

However, there was a gradually increasing NH_3_-N concentration. Figure 7b,d,f and Figure S2 also provide additional supportive results. The ratio NH_3_-N to TN (NH_3_-N/TN) for R_B_ was slightly higher than that of another reactor, which means the hydrolysis rate in organic nitrogen of the former was faster than that of the latter. The paired reactor compartment mean comparison showed a significant difference between B4 and A4, and B3 and A3 (NH_3_-N/TN), with a *p*-value (*p* = 0.00144). In conclusion, we can say the overall hydrolysis rate of R_B_ was better than R_A_, due to the entrapped particulate organics in the compartment “O” during the FW singly-fermenting, where R_A_ was lower.

#### 3.3.2. VFA

VFAs are the most dominant products of the co-fermentation process. Figure 7h shows the variation of total VFA (TVFA) concentration in the effluent of two reactors. For both reactors, the co-fermentation effect could be reflected by the structure characteristic of different acids. It has been known that the products of hydrolysis acidification of organic matter include six short-chain fatty acids (acetic acid, propionic acid, butyric acid, isobutyric acid, valeric acid, and isovaleric acid), but also other small molecules such as ethanol, lactate, and formate, which also contribute to the SCOD values and affect the pH. During all operations of two reactors, six VFAs were measured: acetic acid, propionic acid, butyric acid, isobutyric acid, valeric acid, and isovaleric acid, as well as four representative VFAs: acetic, propionic, butyric, and valeric acids, which are shown in Figure 7i for our discussion. For R_A_ and R_B_, the acetate contributed the highest percentage in the VFAs, 35% and 39%, respectively. The means comparison test showed a significant difference between reactor A and B with a *p*-value = 1.51256 × 10^–5^ (Supplementary Materials Figure S3).

On the basis of the above-mentioned comparative analysis of TVFA, it can be found that, for the two reactors, the significant difference did not appear in the VFA components of effluent but the TVFA concentration. However, the higher TVFA concentration of R_B_ can easily be understood. It has been known that the acidification of FW (60% organics of feedstock) is the primary source of VFA generation in the reactor due to the high content of organic matter [29].

As shown in Figure 7g, the pH in compartment O continued to decrease from the start-up R_A_ and slight stabilized at 5.5 with a slow variation. A pH of roughly 5.5, according to Srisowmeya et al., (2020), might lead to the dominance of acetic acid generation during fermentation [30]. Since it started settling at around pH 5.5, the compartment “O” section of R_A_ looked to be reaching an ideal steady state for fermentation of food waste to acetate. Many solid particles in the food waste crushing solution were caught and precipitated at the bottom of compartment “O”. This precipitate occupied a large proportion of organic matter in the comminution solution. As a result, the amount of organic matter flowing into A1 was lower than it should have been. Therefore, the hydrolytic acidification of the organic matter going into the A1 compartment also had less of an effect on the pH in the compartment A1. In addition, even if the pH of the supernatant in the compartment “O” was low, the volume of supernatant that flows into A1 was not enough to change the high pH of toilet wastewater in the A1 compartment (Figure 7g). A1 had a higher pH value than chamber B1.

### 3.4. The Benefit of Onsite Co-Fermentation of FW and TW for Rural Areas According to Decentralized Sanitation Concepts

There is no universal decentralized sanitation and reuse concept fitting for all situations. Rural area is characterized by farmland activities, having fewer economic resources and random household territorial planning. Usually, the co-stabilization of TW and FW by the enhanced septic tank can be a prudent way to rapidly advance environmental sanitation in developing rural areas.

The anaerobic reactor in this study is designed as an inexpensive and extremely simple operation and maintenance pre-treatment reaction, which is followed by an aerobic reaction. This pre-treatment process only requires the hydrolysis of particulate matter and large organic matter into small or soluble organic matter that can be removed by adsorption in the subsequent aerobic reactor. In order to keep the cost of the wastewater treatment system as low as possible for the rural area, the choice of the technology is delicate task.

This study provided a possibly implemented technology in rural areas for onsite co-digestion treatment of TW and FW. It also makes the nutrient recovery from TW and FW feasible. Moreover, the additional costs for transporting such wastes to the centralized treatment systems are saved since the wastes produced from household buildings can be managed onsite [31,32].

## 4. Conclusions

This study investigated the characteristics of human excreta (HE) and food waste (FW) and their respective fermentation in a typical farming household in China. In addition, two setups of a co-fermentation process for HE and FW were also explored on the basis of the mastering characteristics.

The study concluded that a healthy adult produced an average of 163 g (57.3 gCOD) of feces, 1.6 L (6.7 gN/cap/d) of urine, and 250 g (35.0 gCOD) of FW per day. HE contributes 75% of the nitrogen and phosphorus nutrients, excluding the FW shredder. The mixture of feces and urine was found to accelerate the hydrolysis of urea in urine. Moreover, it needs At least three days for the FW shredding liquid to reach a complete sedimentation equilibrium phase. The removal efficiency of COD at the equilibrium is about 75%. Final, mixing HE with FW after discharge, i.e., the initial unit of the process, was 20% more efficient in fermentation than mixing after the respective pre-fermentation.

## Figures and Tables

**Table 1:** Properties of fresh feces and urine.

	Volume (L/cap/d)	Mass (g/cap/d)	Water Content (%)	Organic Matter (%)	Density (g/mL)	SCOD/COD (%)	pH
Feces	-	163 ± 3	76.9 ± 0.8	90.1 ± 0.6	-	18.9 ± 0.7	7.0
Urine	1.6 ± 0.2	-	-	-	1.0 ± 0.1	94.2 ± 4.4	6.8

## Data Availability

Not applicable.

## Supplementary Materials

The following supporting information can be downloaded at: https://www.mdpi.com/article/10.3390/ijerph19084731/s1, Figure S1. The significance between different compartment for same and different reactor for the ratio SCOD/COD. Figure S2. The significance between different compartment for same and different reactor for the ratio NH_3_-N/TN. Figure S3. TVFA_COD_ mean value statistic. Figure S4. Appearance variation of samples at different temperatures and at 1st day, (a) 4 °C, (b) 20 °C.
