# Supplementary material for: Human Excreta and Food Waste of a Typical Rural Area in China: Characteristics and Co-Fermentation
Source: Int J Environ Res Public Health. 2022 Apr 14;19(8):4731. doi: 10.3390/ijerph19084731 (PMC9028711; doi:10.3390/ijerph19084731)
Supplement: Supplementary file 1 [file ijerph-19-04731-s001.zip › ijerph-1633627-supplementary.pdf]

Supplementary Materials

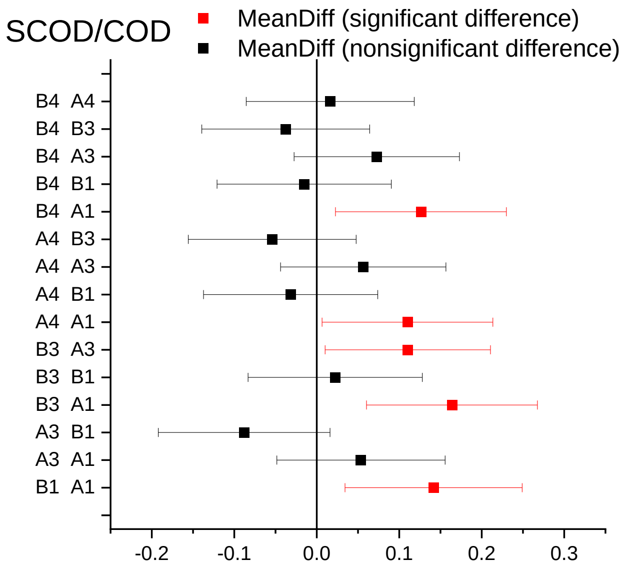

Figure S1. The significance between different compartment for same and different reactor for the ratio SCOD/COD.

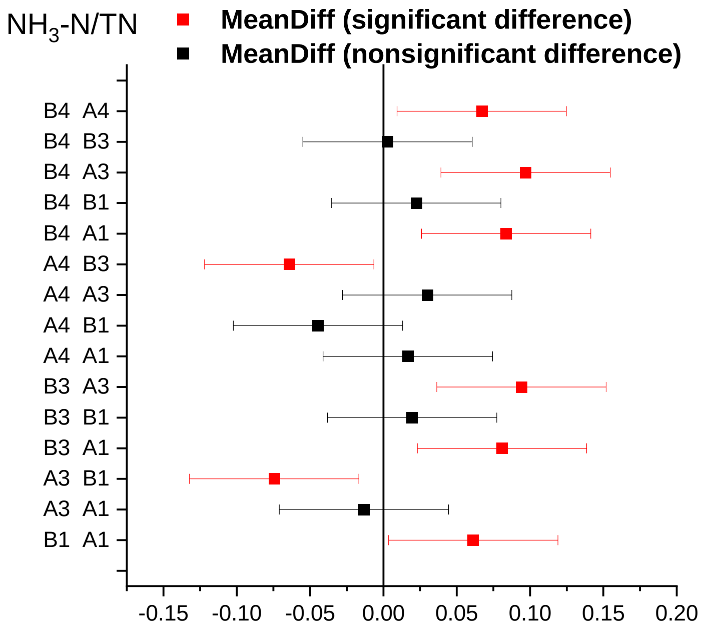

Figure S2. The significance between different compartment for same and different reactor for the ratio NH_3-N/TN.

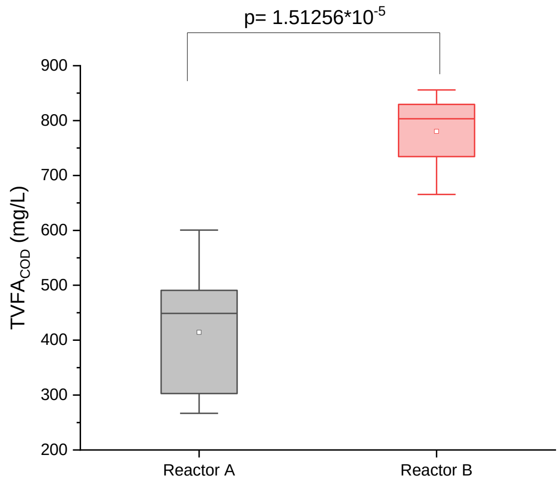

Figure S3. TVFA$_{COD}$ mean value statistic.

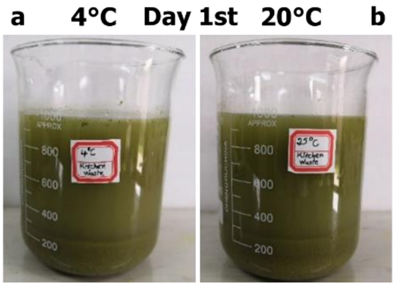

Figure S4. Appearance variation of samples at different temperatures and at 1st day, (**a**) 4 °C, (**b**) 20 °C.
